# Inorganic Nanomaterials Used in Anti-Cancer Therapies:Further Developments

**DOI:** 10.3390/nano13061130

**Published:** 2023-03-22

**Authors:** Olga Długosz, Wiktoria Matyjasik, Gabriela Hodacka, Krzysztof Szostak, Julia Matysik, Patrycja Krawczyk, Anna Piasek, Jolanta Pulit-Prociak, Marcin Banach

**Affiliations:** Faculty of Chemical Engineering and Technology, Department of Chemical Technology and Environmental Analytics, Cracow University of Technology, Warszawska 24, 31-155 Cracow, Poland

**Keywords:** nanoparticles, anti-cancer, thermoablation, treating diseases, inorganic nanomaterials

## Abstract

In this article, we provide an overview of the progress of scientists working to improve the quality of life of cancer patients. Among the known methods, cancer treatment methods focusing on the synergistic action of nanoparticles and nanocomposites have been proposed and described. The application of composite systems will allow precise delivery of therapeutic agents to cancer cells without systemic toxicity. The nanosystems described could be used as a high-efficiency photothermal therapy system by exploiting the properties of the individual nanoparticle components, including their magnetic, photothermal, complex, and bioactive properties. By combining the advantages of the individual components, it is possible to obtain a product that would be effective in cancer treatment. The use of nanomaterials to produce both drug carriers and those active substances with a direct anti-cancer effect has been extensively discussed. In this section, attention is paid to metallic nanoparticles, metal oxides, magnetic nanoparticles, and others. The use of complex compounds in biomedicine is also described. A group of compounds showing significant potential in anti-cancer therapies are natural compounds, which have also been discussed.

## 1. Introduction

Cancer has affected living organisms for more than 200 million years. There is evidence of cancer among the ancestors of modern humans. Unlike infectious diseases, parasitic diseases, and other environmental diseases, cancer is not caused by some entity foreign to the human body. In this case, the destruction factors are human cells that have been transformed into pathological organisms or tumour building blocks [1]. Cancers arise from the transformation of healthy cells due to DNA damage. Disorders in genetic material are caused by mutations of single genes or chromosomes. Tumours can also form in a healthy body as a result of uncontrolled proliferation of cells [2]. A tumour that develops in this way increases in size and then reaches and penetrates the walls of blood vessels. It moves through the lymph vessels and settles in the lymph nodes causing metastasis.

Cancer is the leading cause of death worldwide, accounting for 9.6 million deaths in 2017 [3]. According to the International Agency for Cancer Research, the number of people with cancer in 2020 was 19.3 million. Globally, up to 10 million people died as a result of the disease in 2020. There is a constant increase in the incidence of cancer among both women and men. The breast is the organ most frequently affected by cancer in women; second most affected are the digestive organs. In men, the organs that are the most frequently affected are the digestive organs (lungs) [4].

Due to the increasing incidence of various types of cancer, numerous studies are being conducted with the intent to develop a drug or select a suitable treatment method [5,6]. However, the process is very arduous. There are still many aspects of oncology that have not yet been fully developed, and furthermore, each case of this disease is unique and requires individual treatment [1].

Currently, there are four main methods of cancer treatment, which include surgical resection, chemotherapy, radiation therapy, and biological therapy. Surgical resection is one of the most common ways of treating cancerous tissues and organs. However, in addition to surgical intervention, there are many other ways to mitigate the effects of cancer. They are used when surgical resection is not possible due to difficult access to diseased tissue, the presence of multiple and extensive tumour clusters, or the patient’s preference [7]. The main drawback of this method is the negative impact on quality of life due to the devastating side effects during treatment and, most importantly, it is characterized by limited benefits in terms of survival of the treated patient. Radiation therapy uses high-energy radiation to damage, sensitize, or induce the cell cycle in cancer cells. Both chemotherapy and radiation therapy have a general effect and affect normal cells to a similar degree, and thus the whole body of the patient. These side effects require a therapeutic alternative that can be selective for target tissue and cancer cells [8].

In recent years, much effort has been devoted to the development of reactive oxygen species (ROS)-based cancer treatment strategies. Representative approaches include photodynamic therapy (PDT), sonodynamic therapy (SDT), as well as chemodynamic therapy (CDT) and radioiodine therapy (RDT) [9,10,11]. In these methods, ROS generation is triggered by exogenous energy input, such as laser, ultrasound, and ionizing radiation, or endogenous chemical energy, such as intratumoural H_2_O_2_. ROS generated during this treatment can induce apoptosis or necrosis by damaging the cellular components of cancer cells (e.g., lipids, proteins, and DNA). To enable or enhance these “dynamic therapies”, nanoparticles (NPs) with small sizes, large surface areas, and numerous surface defects are often used as therapeutic agents with catalytic activity to facilitate ROS generation [11,12,13,14].

To increase the effectiveness of cancer therapies, near-infrared (NIR) absorbing agents have recently begun to be used to convert light into heat, increasing the lethality of cancer cells. Photothermal therapy (PTT) is a non-invasive and controlled method with minimal side effects that has shown excellent therapeutic effects in many reports. NIR radiation is more weakly absorbed by biological tissues, so penetration depths of several centimetres can be achieved in biological tissues [15]. To make effective use of this type of radiation, photothermal agents that can convert NIR energy into thermal energy are necessary. PTTs are based on the induction of apoptosis or necrosis of cancer cells through heat generated by photothermal agents under irradiation. Cancer treatment in parallel uses exogenous light to convert tissue oxygen into singlet oxygen (^1^O_2_), which then induces necrosis or apoptosis of cancer cells. Due to reduced tissue absorption and scattering, lasers currently used in cancer therapies use light in the second range (biowindow) of NIR radiation (1000–1700 nm), so they exhibit deeper tissue penetration (>1 cm) [16].

In the case of metal nanoparticles, although they exhibit photothermal properties, they have some limitations such as reduced photostability after a long period of laser irradiation and low photothermal conversion efficiency. One of the problems associated with the application of photothermal therapy is the limitation of the size of photothermal agents. The size of photothermal agents used today is significantly large, which shortens the blood circulation time before they are deposited in the blood vessels. An ideal photothermal agent should meet several requirements, such as appropriate nanosize and uniform shape, large NIR absorption cross-section, high photostability, and low cytotoxicity in living systems.

Absorption of radiation at selected wavelengths of selected nanomaterials, in addition to heat emission, can induce the production of cytotoxic substances, such as singlet oxygen (^1^O_2_), which may destroy cancer cells, but this phenomenon has not yet been widely explained in the literature. Measurement of reactive oxygen species (ROS) makes it possible to detect the level of radicals formed during laser irradiation with wavelengths in the NIR range. For this purpose, a nanocomposite is added to a solution with selected compounds, and the sample is exposed to NIR radiation [17]. As a result of the excitation of photothermal materials, production occurs in a wide spectrum of ROS generated (singlet oxygen, superoxide anion, hydrogen peroxide, and hydroxyl radical) in addition to the increase in the temperature of the medium [18].

To develop the material presented in the paper, it was hypothesized that it is possible to develop a nanocostructure—a nanosystem that allows precise delivery of therapeutic agents to cancer cells without systemic toxicity. At the same time, the nanosystem could be used as a highly effective photothermal therapy system [19]. The nanocostructure could consist of already known and partially characterized components: layered double hydroxide (LDH) nanoparticles, Fe_3_O_4_ nanoparticles incorporated into the LDH structure, metal nanoparticles (i.e., Au, Pd, Pt, Ag) and complex compounds of these metals [20,21,22]. When the advantages of the individual components are combined, it is possible to obtain a product that would be effective in cancer treatment. The carrier would be nanoparticles of layered double hydroxides (LDHs). LDHs are increasingly being proposed for biomedical applications. Their biocompatibility, anion exchange capacity, nanometric particle size, high chemical stability, and ability to maneuver pH enable controlled release and permeation of active substances without the deleterious effects of LDHs themselves. At the same time, by selecting the ionic composition of LDHs, they exhibit photothermal properties, so they can be applied in cancer therapies. By modifying the chemical structure of LDHs before introducing Fe_3_O_4_ nanoparticles, it will be possible to enrich LDHs with magnetic properties. The specific surface area of LDH will increase the efficiency of the deposition of metal nanoparticles, i.e., Au, Pd, Pt, and Ag, making it possible to obtain an active layer showing anti-cancer properties [23,24].

The possibility of using structures such as conjugated polymer nanoparticles (CPNs) in anti-cancer therapy should also be mentioned. These are polymers conjugated to compounds with aromatic or alkyl chain structures. The most common use is polyaniline (PANI), polypyrrole (Ppy), polydopamine (PDA), or polythiophene (PT) [25,26]. Polymers constructed in this way exhibit numerous unique properties with the possibility of wide application in various fields. CPNs show great potential for biomedical applications, including precisely in anti-cancer therapy. The high biocompatibility of CPNs allows them to be safely applied when contacted with living tissues. Their good solubility in water gives them an advantage over other structures used in anticancer therapy, such as quantum dots (QDs). CPNs also yield easy surface functionalization [27,28]. The properties of the described structures are very promising for applications in PDT, PTT, and combined PDT/PTT phototherapies, bioimaging, biosensors, and drug delivery. Among the valuable properties of CNPs are their high light-harvesting ability (especially absorbance in the NIR range), photostability, tuneable spectra properties, and high efficiency in converting absorbed light energy into heat. Thanks to such properties, CPNs can amplify the received signal and can participate in the production of reactive oxygen species (ROS) to destroy cancer cells. Fu et al. in their study showed that CNPs exhibit low cytotoxicity and high specificity in in vitro and in vivo studies in phototherapies. CPNs have a great fluorescence quantum field that may increase with the property modification surface [29]. This is a very important feature in bioimaging or biosensors. CPNs can be a great alternative to structures used in anti-cancer therapies.

The performance of CPNs is compared to that of QDs, which also have amazing properties, but there is a bigger problem with regard to their applicability in medicine. QDs are characterized by high and stable fluorescence intensity, and their most frequently indicated application is bioimaging and biosensing. An additional advantage is the wide bandwidth of the excitation wavelength and the narrow emission spectrum. An interesting application proposal for QDs is Fluorescence Guided Surgery (FGS), in which QDs can target tumours/tissues undergoing resection [30]. Depending on the type of structure and the metals of which they are made of, QDs can exhibit toxicity and poor biocompatibility. For example, graphene quantum dots appear to be safer for use [31,32]. Carbon dots (CDs) are very promising materials in anti-cancer therapy. They also exhibit strong fluorescence properties but are well water-soluble and biocompatible. This, combined with their small size, makes them a great alternative for applications in drug delivery, bioimaging, and biosensors [33]. QDs are also finding applications in phototherapy. It has proven itself as a photosensitizer, which is responsible for the production of ROS under light excitation. Its ability to convert light energy into heat has also been confirmed. Such properties allow them to be used in PDT and PTT techniques [34].

The synergistic and antagonistic effects that occur between the carrier, metal nanoparticles embedded within it, and complexes containing of these metal ions used in photothermal cancer therapies are still unknown. Knowledge of the chemical interactions of metal nanoparticles and complex compounds of these metal ions is still limited. There is no answer to the question of whether it is possible to reduce the effect of releasing metal ions from the surface of nanoparticles while increasing the biocompatibility of materials and maximizing their anti-cancer activity. The behaviour of organic-inorganic systems, ligand substitution on the surface of metal nanoparticles, and the preservation of the stability of complexes in the presence of biological fluids are also still unexplored issues.

**Figure 1:**
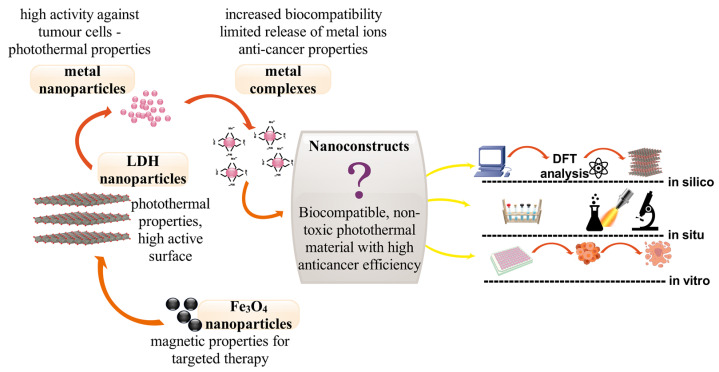
The idea of the nanocostructure and the research cycle envisaged to confirm its properties.

To limit the uncontrolled release of metal ions, nanoparticles can be combined with complexes of these metals. The combination will have a dual function: the release of metal ions that combine with complex compounds will be limited, and the complexes will not be destabilized due to the constant presence of metal ions [35,36]. Figure 1 shows a diagram describing the interactions between the various components of the nanocostructure.

The current state of knowledge does not indicate the use of multi-material targeted complexes of caffeine, quercetin, or cysteine, among others, in combinations with ions and inorganic materials, allowing the use of photothermal therapy with anti-tumour activity. The ability to deliver a therapeutic substance to a specific tumour-affected site would allow the provision of targeted treatment and could increase the effectiveness of the drug [37,38,39].

To the best of our knowledge, there has been no work to date on the interactions occurring between metal nanoparticles and complexes of their ions and the behaviour of their activity in photothermal processes used in cancer therapies. The paper presents numerous results of studies conducted so far, which used nanomaterials and complex compounds and may inspire the development of new, significantly better, and more effective systems for use in anti-cancer therapies. Perhaps it may concern such nanocostructures as the one presented above.

There is a need to present the results of research and theoretical work to acquire new knowledge, which in turn is used to develop technologies for obtaining new nanomaterials, to modify known processes to better control the properties of these new nanomaterials, or to develop such formulations that will not have harmful properties towards living matter. These harmful properties will be reduced while maintaining the functionality of the materials, and the environmental risks associated with their use are also assessed. The goal is to be able to use nanomaterials safely while maintaining their beneficial properties.

## 2. Thermal Ablation

In oncology, the following treatment methods are used: surgery, radiotherapy, chemotherapy, immunotherapy, and hormone therapy. Chemotherapy allows dedicated solutions to be developed, and the branch is developing at a very fast pace [5,6]. The therapy that relies on the use of thermal ablation makes it possible to destroy tumour cells in a controlled manner, without affecting healthy tissue. This article shows how thermal ablation combined with nanotechnology in materials makes it possible to increase the efficacy, safety, and efficacy of the treatment of cancerous tumours.

One of the modern ways of using nanomaterials in biomedicine is the use of gold nanoparticles or magnetic nanoparticles as an intermediary material in thermal ablation of tumour tissues. Compared to conventional methods of therapy, this technique is less invasive and easier to implement. It is also characterized by higher efficiency in destroying malignant tumours. The great advantage of this method is the fact that it is possible to selectively treat a cancerous organ without weakening healthy tissues [7,40]. Many nanoparticles have the ability to absorb various types of energy, e.g., near infrared light (NIR), electromagnetic waves, radio waves, and others. Their ability to convert the energy they gain into thermal energy allows them to be used in thermal ablation processes. The basis of photothermal therapy, in which metallic or magnetic nanoparticles are used, is the excitation of electrons and their subsequent relaxation to the basic level, resulting in the emission of energy in the form of heat. For example, single-walled nanotubes are able to give off heat when exposed to near infrared (NIR). Magnetic nanoparticles are able to generate heat when treated with an alternating magnetic field [41,42]. The generated thermal energy can cause selective necrosis of cancer cells, which makes thermal ablation methods a promising alternative in fighting cancer with minimal invasion of healthy tissues.

In the work of Zhang et al., gold nanoparticles stabilized with polyethylene glycol were used to increase the efficiency of irradiation of HeLa cancer cells in in vitro and in vivo studies. Nanoparticles of various sizes (5, 12, 27, and 47 nm) were used, and the test lasted 24 and 48 h. It was noticed that as a result of irradiation of gold nanoparticles with a size of 12 and 27 nm with gamma radiation, there is a significant decrease in the survival of cancer cells. Nanoparticles with a size of 5 and 47 nm did not show such a strong irritating effect, both during the study of apoptosis and necrosis. The obtained results were also confirmed by the fact that the size and weight of the treated tumour were significantly reduced [43].

Guo et al. conducted work aimed at evaluating photothermal ablation of tumour tissues using gold nanoparticles deposited on an iron oxide core. The aim of the work was to show that nanoparticles are phagocytosed by pancreatic cancer cells (PANC-1), thanks to which they can act as an intermediary increasing the efficiency of thermal ablation. Nanoparticles with a diameter of 30 nm at a concentration of 0, 25, or 50 µg/mL were applied in the study. After directing the nanoparticles to cancer cells, some of them were treated with laser radiation (7.9 W/cm^2^, 5 min), and some were not. Temperature changes were measured, and the number of viable cells as well as proliferative capacity were assessed. Based on the developed temperature curves, it was observed that the increased uptake of nanoparticles, which were then irradiated, resulted in an increased temperature, even by more than 79 °C (for 50 µg/mL). The proliferative capacity of the cancer cells decreased from 100% (for the reference sample) to 2.3%. Research shows that the use of nanoparticles in thermal ablation can be an effective way to treat malignant tumours [44].

## 3. Nanotechnology

### 3.1. Metal Nanoparticles

**Figure 2:**
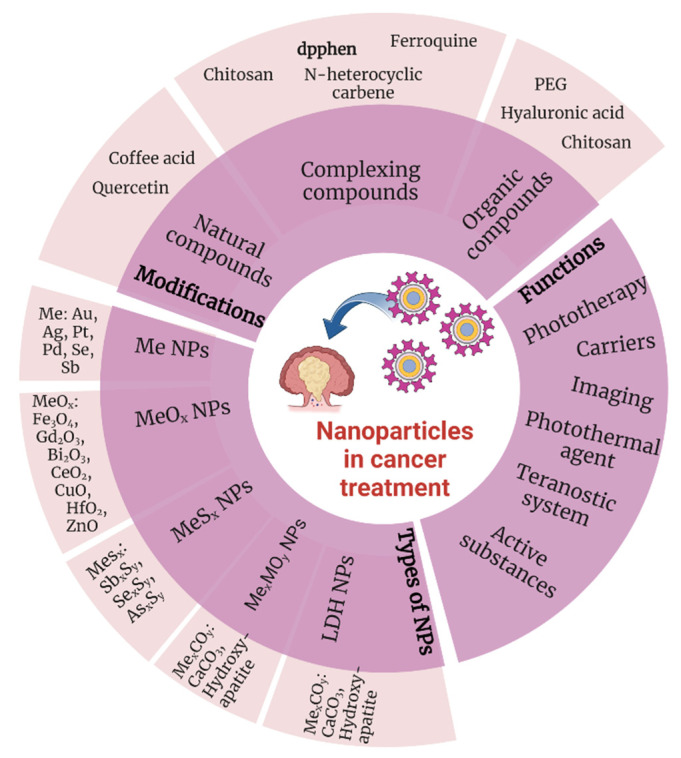
Chemical substances and their functions in cancer treatment (Created with BioRender.com, access: 27 February 2023).

Nanotechnology is one of the fastest growing branches of science with great potential. Nanomaterials, which are used in various application fields, including biomedicine, electronics, construction, and food industry, have also found applications in the fight against cancer. The small size of nanoparticles (<100 nm) and their specific surface area allow various types of functional molecules, such as active substances, to attach to them [45,46,47]. Compared to self-existing therapeutic substances, the resulting systems are characterized by longer life, resistance, and effectiveness as well as having additional functional properties (Figure 2).

Precious metal nanoparticles, particularly palladium, platinum, silver, and gold, are attracting scientific interest because of their unique properties. Among them are high chemical stability or specific optical properties related to the phenomenon of Localized Surface Plasmon Resonance (LSPR), which is significantly influenced by the shape of nanoparticles and the metal they are made of. Noble metal nanoparticles are used as photosensitizers in photothermal anti-cancer therapy, as drug and gene carriers, and in bioimaging [48,49].

Nanomaterials of greatest importance are metal nanoparticles, including Au, Ag, Cu, Pt, and Pd. Gold (Au) with various nanostructures such as nanowires, nanoshells, nanocages, and nanospheres are widely used in the photothermal therapy of cancer cells [50]. Furthermore, Au nanoparticles combined with silver (Ag), in the form of bimetallic Au–Ag nanoparticles [51] and Au/Ag nanostructures with dendrite morphology and hollow interior [52] have shown significant potency in destroying cancer cells in vivo.

**Figure 3:**
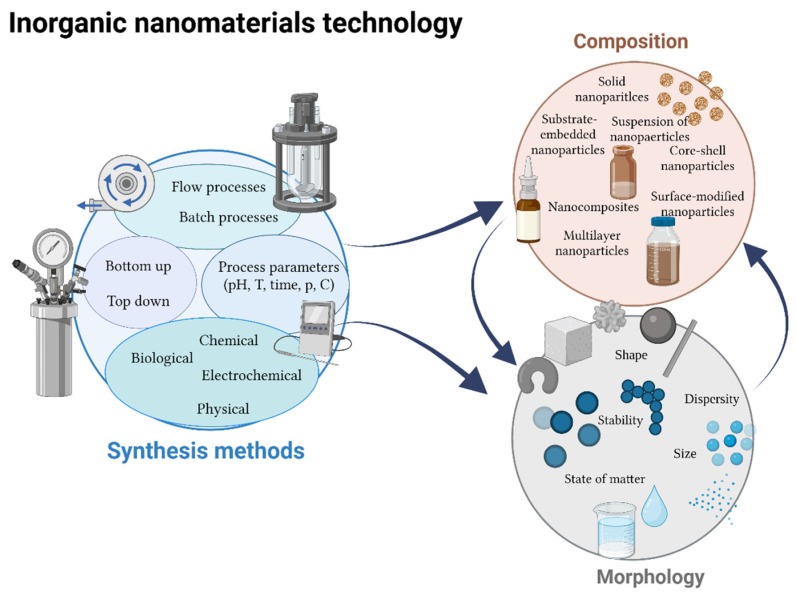
Influence of inorganic nanoparticles preparation methods on their physico-chemical properties (Created with BioRender.com access: 10 March 2023).

By selecting methods to obtain nanoparticles and nanocomposites, it is possible to receive materials selectively acting on targeted cells while remaining non-toxic to healthy cells. This can be conducted both chemically, by selecting reducing agents and system stabilisers, nanoparticle precursors, reaction conditions, and physically by electrochemical and biological methods. Such a wide choice of methods makes it possible to obtain structures of virtually any composition, shape, and size [53,54]. Simultaneously, this approach makes it difficult to systematise the methods that have been gathered over the years. The selection of the right system is frequently carried out experimentally, making it time- and cost-consuming [55]. Figure 3 presents a classification of inorganic nanoparticles in terms of their formation and physicochemical properties.

Currently, the use of plasmonic gold nanoparticles for cancer diagnostics and photothermal therapy due to the intriguing optical properties of these nanoparticles is a popular area in nanomedicine [56]. Advances in synthesis have contributed to the development of AuNPs of various shapes and structures, which exhibit a significant shift toward infrared properties that increase their value in photothermal cancer therapy. Strongly enhanced radiation properties such as absorption, scattering, and plasmonic field make them extremely useful in cancer imaging. Due to their noncytotoxic properties, Au-based nanomaterials are used mainly in photothermal therapy [57,58]. In addition, gold nanoparticles are used as drug carriers, in bioimaging, controlled release of anti-cancer drugs, radiation therapy, photodynamic therapy, and immune therapy [50,55]. AuNPs are being intensively studied in anti-cancer applications due to their ease of synthesis and surface modification, highly enhanced and tuneable optical properties, and excellent biocompatibility that allows medical applications.

Platinum in the form of nanoparticles (Pt NPs) is one of the precious metals that exhibits dual functionality dependent on platinum solubility. When used in soluble forms, they generate DNA strand breaks [59], while their insoluble forms have significant antioxidant capacities [60,61]. For this reason, pure metallic Pt nanoparticles are being investigated for potential application to new anti-cancer methods [62].

Like other nanoparticles, platinum that circulates in the bloodstream can also exhibit toxicity to normal cells, depending on their choice of shape, surface nature, size, and chemical composition of the particles. Numerous publications have investigated Pt nanoparticles size- and shape-dependent compatibility with cells. Manikandan et al. determined the optimal size of Pt nanoparticles for effective photothermal treatment of Neuro 2A cancer cells. Platinum nanoparticles with sizes in the range of 1–21 nm were tested for effectiveness in use as a photosensitizer against Neuro 2A cancer cells. The analysis compared particle sizes ranging from 1 to 18 nm and compared particles of spherical, cuboidal, oval, and floral morphology particles. Even at concentrations as high as 25 and 50 nm, 5–6 nm nanoparticles with a cuboidal shape exerted the desired effect. The number of dead cells resulting from treatment with Pt NPs was then similar to that of the control. A continuing problem is the inability to predict which systems will exhibit the desired properties, including with other cancer cells [63]. In another study, Pt NPs in the size range of 20 nm, 100 nm, and >100 nm with the non-cytotoxic polymer b-cyclodextrin were tested for their effects on cancer cells, and the results revealed that Pt NPs of 20 nm showed greater interactions compared to larger particle sizes [64]. A limitation of using particles smaller than 20 nm is the increased likelihood that Pt particles accumulate in internal organs and pass through the gastrointestinal tract [65]. Although there are reports on the effects of Pt nanoparticles on DNA, to date, there are no direct studies correlating the effect of size and shape dependence on the toxicity/compatibility of Pt nanoparticles against cancer cells [66].

In addition to the use of nanoparticles as carriers of active substances and as tumour cell degrading agents, nanoparticles are also used in thermoablation processes. Due to the target application of thermoablation in vivo systems for cancer treatment, materials are being sought that would lead to an increase in temperature of up to 45 °C, which is sufficient to trigger the apoptotic pathway. This is one of the main advantages of using Pt NPs as a photothermal agent. Cells treated with nanoparticles without a laser often show no effect on cell morphology. Only the combination of the laser treatment method in the presence of Pt shows a high ability to kill cells such as Neuro 2A [62].

Among the known metal nanoparticles, in addition to Au and Pt, Pd NPs are frequently studied. In addition, for Pd nanoparticles, the shape, particle size, and surface modifications of NPs are important. Palladium nanoparticles with a spherical shape, a size of 5 nm, and good dispersion were studied against ovarian cancer cells. The effect of nanoparticles on tumour cell viability was studied, and as a result, Pd NPs were shown to dosing-dependently reduce cell viability. In particular, doses higher than 6 μg/mL induced significant cell death. The effect on cell morphology was also studied-at 6–10 g/mL, Pd NPs induced noticeable morphological changes, which were more severe as the concentration of Pd NPs increased. In addition, cells showed shrunken cell membranes, and cell viability and shape were altered [67]. Some studies have reported the possible toxic effects of Pd NPs and their propensity to induce adverse health effects, such as concentration-dependent cytotoxicity, apoptosis, and changes in the release and expression of numerous cytokines [68,69,70].

The impressive antimicrobial activity of Ag NPs provides a starting point for the design, development, and implementation of new and performance-enhanced nanosilver-based biomedical products, such as anti-cancer agents, drug delivery platforms, orthopedic materials and devices, bandages, antiseptic sprays and catheters, dental fillings, or as a component of bone implants. In particular, Ag nanoparticle-based nanosystems have been evaluated as suitable carriers for various therapeutic molecules, including anti-inflammatory, antioxidant, antimicrobial, and anti-cancer biologics [71,72].

Similar concerns apply to the use of silver nanoparticles, which despite their high bactericidal, virucidal, fungicidal, ROS-generating activity, relatively low price (compared to other metal nanoparticles), and satisfactory stability over time, have proven toxic properties. This makes it difficult to precisely act on cancer cells without interfering with healthy cells. Consequently, silver is not widely used in nanoparticle-based drug delivery applications, and it is replaced by gold or other nanomaterials [73]. To a large extent, the toxicity of silver depends on its dose. Toxicity studies conducted on a rat ear model have shown that exposure to Ag nanoparticles results in significant mitochondrial dysfunction, followed by temporary or permanent hearing loss, depending on the dose. Even low concentrations of Ag NPs were absorbed by retinal cells and caused significant structural abnormalities, due to the increase in the number of cells that underwent oxidative stress [74]. The toxicity of Ag nanoparticles is related to their transformations under biological conditions and in environmental media, including their interactions with biological macromolecules, surface oxidation, and the release of silver ions. It is also very important to precisely distinguish the degree of toxicity associated with both nanosilver and ionic silver [75].

On the other hand, Ag nanoparticles can fuse with and easily penetrate mammalian cells [76]. A valuable advantage of Ag NPs is their specific fluorescence, making them suitable candidates for detection and dose escalation purposes in X-ray irradiation applications [77]. Silver nanoparticles are also plasmonic structures, capable of specifically scattering and absorbing light incidents in specific areas. Once selectively absorbed by cancer cells, the scattered radiation from Ag nanoparticles can be used for imaging purposes, while the absorbed radiation can be used for selective hyperthermia [78]. Silver nanodrops have been investigated for use in photothermal therapy and in vivo imaging with CT scans. Albumin-stabilized Ag nanodrops (ANDs) with ultrathin size (approximately 5.8 nm) and strong X-ray attenuation (5.7313 HU per mM Ag) were obtained. Silver nanoparticles are excreted directly from the body through urine, so they can be used as CT imaging agents and as effective photothermal therapy agents. Tumour growth inhibition reached 90.2% after photothermal therapy with Ag nanoparticles. Silver nanoparticles showed little toxicity to oral epithelial cells-cell viability was over 85% even at a concentration of 500 μg/mL. Megascopic hemolysis was not observed, thus indicating good compatibility of ANDs with blood [79]. In addition, cells exposed to a NIR laser with silver nanoparticles had a high death rate. This indicates that ANDs can effectively destroy cells when used with the NIR laser. The temperature of the tumour surface under NIR irradiation increased rapidly from approximately 32 to 52 °C, which was sufficient to induce hyperthermia and kill cancer cells. In the groups treated with silver nanodrops (with or without irradiation), there was no significant organ damage or inflammation in all normal tissues, such as the heart, liver, spleen, lung, and kidney, confirming the very low toxicity of silver nanodrops in vivo at the doses tested [78].

Bismuth is an element with a high atomic number (Z = 83) that is reasonably inexpensive, available in large quantities, and most importantly, biocompatible. This makes it a good candidate for research as a radiosensitizer, i.e., a compound that makes cancer cells more sensitive to radiotherapy [80]. Depending on the method of synthesis, bismuth oxide can scavenge reactive oxygen species (ROS), resulting in increased cell proliferation, or generate ROS, which in turn results in high cancer cell mortality [81]. Because of the regulated cytotoxicity and real-time imaging, it is a promising candidate for a theranostic system. At the same time, bismuth oxide and other bismuth oxide-based compounds are biocompatible and have been widely used in many medical and cosmetic applications for many years [82].

Information on other nanoparticles used in anti-cancer therapies is summarized in Table 1. The morphology of the nanoparticles, the presence of additives that functionalize their surface, as well as the functions they performed in the described studies, are compared.

### 3.2. Metal Oxide Nanoparticles

Oxide nanoparticles, mainly Bi_2_O_3_, ZnO, TiO_2_, CuO, CeO_2_, HfO_2_, and Gd_2_O_3_, are an alternative to the use of metal nanoparticles [2,158,159]. Metal oxides exhibit different properties compared to MeNPs and are often less reactive and less toxic to living cells, and because of their increased active surface area, they can act as carriers of active substances (Table 2).

However, metal oxide nanoparticles also have some disadvantages. Among those, one of the main ones is that macrophages of the phagocytic system are capable of rapidly eliminating them even before they reach the site of cancer cells. Another limitation associated with the use of oxide nanoparticles in active substance transport systems is the possibility of aggregation of these materials, resulting in a significant increase in particle size [160]. To counteract particle aggregation, compounds that modify the surfaces of Fe_3_O_4_ nanoparticles are added to ensure the stability of their nanosize. At the same time, it is important to increase their biocompatibility and to enable controlled drug coupling on their surface.

Due to their versatile properties, Fe_3_O_4_ nanoparticles are used, among others, in medicine, where they have been used for years in anticancer therapies, e.g., as a carrier of therapeutic substances or as a hyperthermia inducing agent. This phenomenon is particularly important because nanoparticles can selectively affect cells. As a result of hyperthermia, a process of mildly raising the temperature to approximately 40–43 °C, and tumour cell death are induced. This enhances the effect of radiotherapy and chemotherapy [161,162]. This effect can be strengthened by activating tumour localisation with near-infrared (NIR) light. These properties have been successfully applied in the development of modern photothermal therapies. Nevertheless, several important issues limiting the potential of Fe_3_O_4_ nanoparticles still need to be overcame. These are limited selectivity of action on cancer cells as well as on healthy cells or efficiency of action and possibility of toxic effects of the particles on the body [26].

Over the past few years, remarkable progress has been made in the development of multifunctional systems for cancer therapy. Combining three or more different strategies in a single system is now becoming a representative challenge in the biomedical field, and many researchers are paying attention. One component of such integrated systems may be magnetic properties, such as Fe_3_O_4_ nanoparticles, which have found interest in drug delivery systems. Fe_3_O_4_ nanoparticles have excellent properties for use in oncology treatment systems due to their unique structural parameters, size, and external features [163]. Interest in the use of magnetic nanoparticles has also increased because of the possibility of precisely targeting them to the tumour site using an external magnetic field, thus reducing the accumulation of nanoparticles in unwanted areas and thus reducing negative effects in other parts of the body, which is a significant drawback of other classical therapies, such as aforementioned chemotherapy and radiotherapy. An interesting property of these magnetic nanoparticles (Fe_3_O_4_) is their ability to heat up when exposed to a magnetic field. The heat generated is the result of the rotation of magnetic spins in the particle (Néel relaxation), or the rotation of the particle as a whole (Brownian relaxation). The heat generated in this way can be used in cancer treatment, so these nanoparticles could play an anti-cancer role on their own [7]. A paper by Hilger et al. tested a new intervention procedure to treat breast cancer in vivo. The method involved the introduction of iron oxide particles into tumour tissue and its subsequent exposure to an alternating magnetic field. Human mammary gland adenocarcinoma cells were implanted into mice, and then 4 or 18 mg of iron oxide per 100 mg of tumour tissue was injected intraperitoneally. After 20 min, the animals were exposed to an alternating magnetic field for 4 min. During the study, the temperature in the tumour area and the histological evaluation of the treated tissue were measured. A temperature increase of 12–73 °C was recorded, and histological examinations showed the presence of early stages of tumour cell necrosis. The authors conclude that the proposed method makes it possible to produce local thermal sites in the tumour area, which results in necrosis of cancer cells [7]. Core-shell CoFe_2_O_4_@MnFe_2_O_4_ nanomaterial for treatment of liver cancer in-silico, researched by Suleman et al., was the material used similarly. Firstly, nanomaterial was injected into liver tumour model with 1.3 mm width needle, and later, the magnetic field was applied. Amplitude of used magnetic field was 2.65 kA/m and 500 kHz. Within 10 min of irritation, tissue was heated up until 45 °C, and later on, it was kept in the tissue until the end of the experiment for 60 min. Tumour damaged was predicted by integral model, and accordingly, 63–99% cancer cells were damaged [164]. Furthermore, these ferric structures may be used in corporation with cobalt, nickel, manages, zinc and their alloys or oxides. Aforementioned particles can be used successfully with photoablation therapy method [165].

The ZnO and TiO_2_ nanoparticles, due to their large surface-to-volume ratio and the large gap between their conduction band and the valence band, show unique properties, such as better light absorption and very good catalytic and photocatalytic properties [166]. ZnO nanoparticles, compared to TiO_2_, exhibit greater cytotoxicity to cancer cells through the generation of reactive oxygen species (ROS), causing oxidative stress and ultimately cell death [158]. ZnO NPs can also reduce cell viability by altering the integrity of the membrane and damaging the cell DNA structure [167]. Zinc oxide nanoparticles are also a promising component of an active substance delivery system due to their highly developed surface area, wide availability, low toxicity, and stability [168].

Cerium oxide nanoparticles, due to their unique properties, such as their ability to absorb ultraviolet (UV) radiation, high thermal stability, high hardness, high oxygen storage capacity, and the ability to rapidly change the degree of oxidation between Ce(III) and Ce(IV), are a common part of research. Cerium oxide nanoparticles in various sizes and crystal structures have antioxidant properties similar to the enzymes superoxide dismutase and catalase [169]. CeO_2_ NPs are characterized by selective toxicity in cancer cells, yet low toxicity to healthy cells. In the natural cellular environment, they act cytoprotectively, where they act as antioxidants. However, in the acidic pH environment which occurs in cancer cells, they act strongly oxidatively and exhibit strong cytotoxic effects. They can lead to cancer cell death through a series of changes in intracellular redox activities and peroxidative stimulation [170]. Cerium oxide nanoparticles have shown the ability to sensitize pancreatic cancer cells to radiation without adversely affecting the viability of healthy cells or their sensitivity to radiation. Nano-CeO_2_ during radiation therapy has also been shown to selectively increase the production of reactive oxygen species in pancreatic cancer cells [171] and help protect cells in healthy tissues from radiation-related damage [172,173].

CuO nanoparticles, because of their versatile catalytic, photothermal, photoconductive, antimicrobial, and anti-cancer properties, are materials with high application potential. Furthermore, copper oxide nanoparticles have limited toxicity, while exhibiting high chemical and physical stability and long-lasting activity compared to organic antimicrobial agents [174]. The anti-cancer activity of nano-CuO has been demonstrated preclinically in various types of cancer, including liver cancer, lung cancer, breast cancer, cervical cancer, and pancreatic cancer [175]. The mechanism of anti-cancer action of copper oxide involves the efficient production of reactive oxygen species, which induce oxidative stress, leading to DNA damage and ultimately cell death via apoptosis. The nanoparticles also have the previously mentioned effective antibacterial and antifungal activity and can serve as a contrast agent for multimodal imaging (both ultrasound and MRI).

Approximately 35% of all MRI examinations are performed with contrast agents, and Gd(III) complexes are by far the most widely used contrast agents in clinical practice. With seven unpaired electrons, Gd(III) has a large magnetic moment (7.94 µB) and has slow electron relaxation times (approximately 10–9 s) at magnetic field strengths used in MRI techniques [176]. A significant drawback of these materials is the high toxicity of Gd ions washed out of Gd_2_O_3_ nanoparticles, which has encouraged researchers to search for new stable and safe gadolinium-based contrast agents capable of selective localization in tumour tissues. Liposomes, micelles, and polymers containing suitable chelating agents have been identified as a promising strategy for controlling Gd ion leakage and toxicity [177]. Recently, ultrasmall gadolinium oxide nanoparticles (Gd_2_O_3_) coated with diethylene glycol (DEG) as a surface ligand have been shown to be effectively internalized and tolerated by THP-1 cells. This is a human monocytic leukaemia cell line cultured in suspensions that is well known for its phagocytic behaviour. As a result, the labelled cells are brightly on MRI images [178].

Hafnium oxide nanoparticles are already widely used in cancer therapies around the world, the market representative being, for example, the NBTXR3 product. This material is administered as a single injection into the tumour prior to radiation therapy. NBTXR3 is inert before radiation, but during RT treatment, NBTXR3’s high electron density allows for a high probability of interaction with incoming ionizing radiation, increasing energy dose deposition in cells. Due to its physical mode of action, NBTXR3 activated by radiotherapy is expected to be effective in all types of solid tumours. Therefore, NBTXR3 may lead to better efficacy with the same radiation dose or maintain similar efficacy with a lower radiation dose [179,180,181,182].

### 3.3. Layered Double Hydroxides (LDH)

Over the past few years, remarkable progress has been made in the development of multifunctional systems for cancer therapy. The combination of different strategies in a single system is now becoming a representative challenge in the field of biomedicine, which many researchers are paying attention [194]. Compounds with developed surfaces, usually metal oxides and inorganic salts, including hydroxyapatites and calcium carbonates, are the basis of nanosystems. The literature also describes applications of other compounds, eliminating the disadvantages of commonly used carriers, such as LDH (layered double hydroxides). The nanometric particle size, biocompatibility, anion-exchange capacity, high chemical stability, and ability to manoeuvre pH enable controlled release and permeation of active substances without causing harmful effects of the nanoparticles themselves (Table 3). The high immobilization capacity of active substances is due to both the layered structure of LDH and the presence of a charge on the LDH surface. As a result, it is possible to intercalate LDH with active substances. At the same time, as pH-responsive compounds, LDHs are a suitable component of selective systems for use against cancer cells [195].

The delivery of active ingredients via nanocarriers is driven by the need to increase the stability of active ingredients, especially those with limited water solubility, and to induce the controlled release of therapeutic drugs. Positively charged LDH can be easily internalized into the negatively charged cell membrane, and protonation of -OH groups around metal ions allows LDH to release intracellular drugs in response to the acidic tumour microenvironment [196,197]. LDH nanoparticles, as inorganic materials with a highly developed surface and layered structure and as nanocarriers of active substances, will enable the system to reduce toxicity while providing a high capacity for them. In most cancer tissues, the pH is similar to that of lysosomes or endosomes (pH < 6.0) and lower than that in healthy cells (pH = 7.4), therefore the use of pH-responsive materials will reduce unwanted drug release in the blood and improve drug release at targeted tissue. Such nanocarriers release the drug more precisely at diseased sites than in the immediately surrounding healthy cells [198].

Examples of metals incorporated into LDH while exhibiting improved absorption of NIR radiation include cobalt, iron, and copper hydroxides [185,186]. Alternatively, Zn-Al-LDH or Mg-Al-LDH can be modified by modifying their structure with transition metal ions [199]. Intercalation of the ions in the interlayer spaces increases the absorption of energy in the sample, allowing for extended storage and gradual release. Due to supramolecular interactions between LDH and ions embedded in the LDH structure, the photothermal conversion efficiency is increased [200].

## 4. Organic–Inorganic Complexes

Organic–metallic complexes used in medicine can have different functions in anti-cancer therapies, due to the type of particle sizes obtained [200]. Organic compounds (arsenic, carbonaceous, carbonyl and proteins or polysaccharides) in combination with metals are used. Complex systems are chosen for the biocompatibility of the particles to enhance drug uptake inside the altered cells. The presence of compounds of natural origin or recognised by cells as harmless is an important element of research into compounds with potential anti-cancer applications [15]. When combined with metals, complex compounds show enhanced action in catalysed reactions, affecting cell metabolism by limiting harmful processes [16]. However, many of the reaction mechanisms are not known, and the results for individual compounds vary considerably. In addition, for the various complexes, there are no complete data on their effects on cancer cells. The complexes studied should be compared by matching the best performance and multifunctional potential of the materials, with which new cancer treatment strategies can be developed [15,16].

The literature also contains studies on the use of organic–inorganic complexes in the treatment of cancer. Bertrand et al. obtained a series of new complexes of gold and N-heterocyclic carbene (NHC) with an attached xanthine ligand. The study tested the effectiveness of the produced compounds for their antiproliferative properties in human cancer cells and non-cancer cells in vitro, as well as for their toxicity in healthy tissues ex vivo. It turned out that the bis-carbene complex showed selective activity against human ovarian cancer cell lines, with a weak toxic effect on healthy organs [204].

A study by Fabbrini et al. concerned a silver(I) N-heterocyclic carbene (NHC) complex of bis(1-(anthracen-9-ylmethyl)-3-ethylimidazol-2-ylidene)silver ([Ag(EIA)_2_]Cl). The complex was found to be stable in organic solvents and in physiological fluids. It exhibits pronounced cytotoxic activity in vitro against human SH-SY5Y cells. It was found that the in vitro antitumour activity is apparently related to the level of internalization of the drug. It is noteworthy that [Ag(EIA)_2_]Cl does not react with several model proteins but is capable of binding the C-terminal dodecapeptide of thioredoxin reductase hTrxR(488-499) and strongly inhibiting the activity of this enzyme. Internalization involves covalent binding of one or two carbene ligands to the C-terminal dodecapeptide with simultaneous release of the silver cation [205].

The work of El-Tabl et al. presents new organic-metallic complexes constructed from a metal (II) bound to the H2L ligand, i.e., 2-hydroxy-N′-((Z)-3-(hydroxyimino)-4-oxopentane-2-ylidene)benzohydrazide. The complexes produced were characterized using a distorted octahedral geometry. An assay of the cytotoxic activity of the ligand and its complex with the metal showed potent cytotoxic effects against human liver cancer growth (test on HepG2 cell lines) compared to the clinically used Sorafenib (Nexavar) [206].

In Table 4, data on different complexes with their anti-cancer activities have been collected. Unfortunately, these data are very different for each of the materials, and many of them do not produce a holistic effect on tumourigenic lesions. They are difficult to apply to a selected site in the body, due to their low selectivity [80].

## 5. Conclusions

Bearing in mind the well-being of oncological patients, as well as the dynamic progress in modern anti-cancer therapies, it is extremely important to search for new methods of cancer treatment. This review presents the most modern techniques for treating diseased tissues with the use of a new generation of equipment and materials. Undoubtedly, metal complexes, compounds of natural origin, and metallic and oxide nanoparticles have great potential in the delivery systems of active anti-cancer substances and have shown anti-cancer activity themselves.

The use of photothermal therapy in cancer treatment seems to be an appropriate direction to reduce the development of diseases, but it is unknown how the increase in temperature and the formation of active forms affect the active substances deposited on the surface of photothermal carriers. It is still important to understand in detail the mechanisms affecting the stability and photothermal properties of nanocarriers (e.g., based on magnetic LDHs), metal nanoparticles, and complexes of these metals, as at present, very little is still known on these issues. The task is to design potential substances with synergistic anti-cancer effects, resulting from photothermal and magnetic properties and the release of metal ions into the tumour cell environment. These substances should possess the lowest possible toxicity to healthy cells but high toxicity to cancer cells, resulting from high uptake of ions released in the tumour environment and the formation of highly reactive oxygen radicals in the tumour environment.

In connection with the hypothesis raised in the paper about the possibility of obtaining a nanocostructure that allows precise delivery of therapeutic agents to cancer cells and that could be used as a system with high efficiency of photothermal therapy, the questions remain: How could the efficiency of thermal energy storage by double layered hydroxides in such a construct be achieved? Will adsorption of metallic nanoparticles on the surface of metal oxide-LDH junctions increase their potential effect on cancer cells? The combination of metal nanoparticles and complex compounds of these metals in the construct will make it possible to reduce the release of ions and their harmful effects on healthy cells. Will this affect the stability of the complexes? Will the nanocostructure obtained have a function in anti-cancer therapies, and will it not exhibit toxic properties against normal cells?

However, such an approach to the problem posed has not yet been reported in the literature. Solving the presented problem and obtaining answers to the posed questions will contribute to progress in obtaining new materials and developing anti-cancer therapies. Knowledge of the mechanisms of the above-mentioned chemical processes may be important for controlling the properties of both nanoparticles (by eliminating their cytotoxic effect, resulting from uncontrolled release of ions) and metal ion complexes (ensuring their ionic stability) and their interaction with photothermal compounds of biological significance. NIR radiation can result in the modification of chemical bonds and changes in the oxidation degrees of individual components, which should be limited; hence, research on this topic to obtain detailed information on the underlying processes is needed.

Combating cancer is one of the greatest challenges of civilization in the coming decades. In view of the projected increase in the incidence of cancer and the resulting consequences in terms of high mortality rates and serious social and economic consequences, research on the subject carries great importance. The primary goal accompanying the presented problem is to improve the quality of life of oncological patients by minimizing the side effects of the therapy undertaken. This is because targeted therapy and direct action of the anti-cancer agent at the lesion site reduce the systemic reaction that occurs when using, for example, chemotherapy. From an economic point of view, it should be noted that cancer diseases generate both direct costs (resulting from their diagnosis and treatment) and indirect costs (related to losses in production and service revenues). Exploring the proposed topic will help reduce these negative consequences of cancer.

## Figures and Tables

**Table 1:** Metal and non-metal nanoparticles used in anti-cancer therapies.

NPs	Size/Shape of Nanoparticles	Function	Ref.
Ag	25 ± 5 nm, spherical	Hybrid nanocapsules for drug delivery containing silver nanoparticles on the surface, enabling controlled drug release under ultrasound	[82]
Ag/GO	15 nm (PXRD)	Synergistic antimicrobial effect of AgNPs-ciprofloxacin with reduced cytotoxicity and high stability	[83]
Ag	37 ± 8 nm, spherical	Stable and non-toxic drug carrier	[84]
Ag	20 ± 4 nm, spherical	Stable radionuclide carrier for radiotherapy captured by cancer cells with low toxicity to healthy cells	[85]
Ag/GO	≈20 nm, spherical	Easy to functionalize hybrid drug nanocarrier that also enables SERS bioimaging	[86]
Ag	≈120 nm, nanocages	Biocompatible nanocrystalline material for photothermal therapy	[87]
Ag	≈6 nm, nanodots	Low toxic material with high X-ray attenuation for imaging and drug for photothermal therapy	[88]
Ag/RGO	≈12 nm, spherical	Material for photothermal and photodynamic therapy	[79]
Ag	≈79 nm, spherical	Material with antimicrobial, antioxidant, and anti-cancer activity	[89]
Ag	38–63 nm, cubic/square	Material with anti-cancer effects	[90]
Ag	≈27 nm, spherical	Functionalized material with anti-tumour activity and enhanced biocompatibility	[91]
Ag	5–25 nm, oval and spherical	Material with antifungal and antitumour activity	[92]
Ag	≈23 nm, spherical	DDAPG drug carrier with anti-cancer, antimicrobial and antioxidant activity, with enhanced bioactivity and biocompatibility	[93]
Ag	21–25 nm, spherical	Functionalized 5-fluorouracil drug carrier, pH-sensitive with modulated release, with antitumour and antimicrobial activity	[94]
Ag	11 nm, spherical	Drug carrier with reduced toxicity and anti-tumour effects	[95]
Ag	142 ± 33 nm, spherical	Material with anti-cancer, antimicrobial, antioxidant, and anti-inflammatory activity with reduced toxicity	[96]
Ag	2–24 nm, spherical	Material with anti-cancer and antimicrobial activity	[97]
Ag	≈30 nm, spherical	Material with anti-cancer and antimicrobial activity	[98]
Ag	42 ± 5 nm, spherical	Functionalized anti-cancer material with low cytotoxicity against healthy cells, antimicrobial activity	[99]
Ag	≈72 nm, spherical	Functionalized drug carrier with anti-tumour activity to increase the effectiveness of the drug used	[100]
Ag	≈20 nm, spherical	Functionalized material with anti-cancer activity	[101]
Ag	50–90 nm, spherical	Functionalized material for photothermal therapy with enhanced antioxidant activity, anti-cancer activity, and increased biocompatibility, and low toxicity to healthy tissues (organs)	[102]
Ag	37 nm, spherical shape	Drug carrier in anti-cancer therapy	[103]
Ag	45 nm, spherical and oval particles	Cytotoxic activity (against Human hepatoblastoma cells (Hep G2)) and antibacterial activity	[37]
Ag-Chitosan	72 nm, oligomeric chitosan coated silver nanoparticles	Drug carrier with anti-cancer therapeutic potential	[104]
Ag	20 nm, spherical shape	Anti-cancer activity with dual inhibitory action on COX-2 and NF-jB expression	[38]
Ag-PVP	50–90 nm, spherical shape	Photothermal therapy technique for benign prostate hyperplasia (BPH)	[105]
Au	10–15 nm	Biodegradable material for photothermal therapy, embedded in liposomes	[106]
Au	5–12 nm, spherical	Low toxicity and highly selective gene carrier for cancer therapies	[107]
Au	10–20 nm, spherical	Element of a non-toxic and antioxidant antitumour composite (chemotherapeutic)	[108]
Au	<10 nm, spherical	Stable carrier possible for functionalization with organic selenium compounds, reducing cytotoxicity, and increasing selectivity and efficiency against cancer cells	[109]
Au	≈100 nm, triangular flakes	Functionalized drug carrier active in the presence of ultrasound to enhance the effectiveness of cisplatin against cancer cells resistant to the drug	[110]
Au	≈50 nm, nanoflowers	Photothermal therapy material embedded with polymyxin E (PE) with high photothermal conversion, antimicrobial activity, and low toxicity to healthy tissues	[111]
Au	≈22 nm, spherical	Drug carrier (doxorubicin), increasing the effectiveness of radiotherapy and radiochemotherapy with increased accumulation in the acidic tumour environment	[112]
Au	7 ± 4 nm, spherical	Functionalized material that induces hyperthermia under the influence of light or radiofrequency electric field with high biocompatibility and low cytotoxicity	[113]
Au	NPs encapsulated in two types of cell vesicles (~30 nm and ~4–6 nm membrane thickness)	Inducing an immune response against cancer cells via Au nanoparticles camouflaged with exocytotic vesicles derived from B16F10 cancer cells and CDs dendritic cells	[114]
Au	Spherical, 14 ± 3 nm modified with citrate; ~19 nm modified with PEG350	Photothermal agent, enhancing cytotoxic effect of DOX drug in breast cancer treatment using PTT; effect confirmed against MCF-7 cells with λ = 530 nm 3.44 W/cm^2^ irradiation	[115]
Au	20.5 ± 1.9 nm after modification	Photocrosslinking PEGylated and diazirine-decorated particles for enhanced PTT and photoacoustic tumour imaging, confirmed in female BALB/c mice, 2 mg/L with λ = 405 nm 1 W/cm^2^ irradiation	[116]
Au	12 nm before modification	Cytochrome c-modified pH-responsive photothermal agent, activity confirmed on B16F10 and MDCK-GFP cells with λ = 660 nm 14 W/cm^2^ irradiation	[117]
Au	Various particle shapes and sizes in the range of 7 × 26–400 nm	Particles targeting anti-cancer activity using different mechanisms in PTT and PDT with photosensitizing properties	[118]
Au	9.9–11.6 nm before modification	Photothermal agent using PBS or modified PEG for simultaneous PT/PA and PTT imaging of tumours, confirmed to work on C26 organisms with λ = 808 nm, 1 W/cm^2^ irradiation	[119]
Au	Different shapes and sizes of particles—an overview	Modified gold nanoparticles in anti-cancer therapy—PTT, RFA, drug transport, and modulation of angiogenesis	[120]
Au	Different shapes and sizes of particles—an overview	Modified gold nanoparticles in anti-cancer therapy—PTT, RFA	[121]
Au	1.9–74 nm after various modifications	Nanoparticles as radiosensitizers, physical, chemical, and biological mechanisms	[122]
Au	Different sizes depending on the method of obtaining	Drug carriers, contrast agent in imaging and photosensitizer in PTT, substrate in SERS imaging	[123]
Au	Different shapes and sizes of particles—an overview	Modified carriers of drugs, antibiotics, genes, proteins, molecular nanoprobes for detection and monitoring of target molecules	[124]
Au	Gold nanoparticles embedded on liposomes, 100–120 nm, spherical shape	Killing cancer cells via photothermal therapy	[125]
Au	1.8 ± 0.32 nm after modification	Increased targeting of HIV drug carriers, p-mercaptobenzoic acid modification, effect confirmed on PBMCs, HBMECs, and macrophages cells	[126]
Au	14 nm before modification	Antimicrobial agent capable of surface self-adaptation, modified with a mixture of SAMs and fast responders to pH change, effect confirmed on MRSA bacterial biofilm	[127]
Bi	105 nm after modification)	Photosensitizer, contrast and photoacoustic agent; stabilized DSPE-PEG2000 with proven activity against C6 cells (LC80 200 μg/mL with λ = 808 nm 1 W/cm^2^ irradiation)	[128]
Bi	~42 ± 2 nm, after modification ~50 ± 2 nm	DSPE-PEG2000-stabilized photosensitizer directed and camouflaged by CT26 cell membranes deposited on a surface with proven activity against CT26 cells (LC99 100 μg/mL with λ = 808 nm 1 W/cm^2^ irradiation)	[129]
Bi	40 nm, after modification 56 nm	Radiosensitizer directed and stabilized by folic acid-PEG2000-DSPE, camouflaged by RBCs cell membranes deposited on the surface with proven effect against 4T1 cells (LC80 100 μg/mL with 9 Gy X-ray irradiation)	[130]
Bi	~10 nm, after modification ~300 nm	PVP-modified radiosensitizer with attached anti-corrosive graphene oxide with proven activity against 4T1 cells (IC44 2 mg/mL under NIR λ = 808 nm irradiation, IC59 2 mg/mL under X-ray irradiation, and IC90 2 mg/mL under NIR λ = 808 nm and X-ray irradiation)	[131]
Bi	25 nm, spherical	Potential radiosensitizer, contrast agent with high biocompatibility (tested in mice)	[132]
Bi	3.6 nm, after modification	Radio- and photosensitizing agent with contrast and photoacoustic properties, stabilized by DSPE-PEG2000, directed by LyP-1 peptide with confirmed activity against 4T1 cells (IC mg/mL on NIR λ = 1064 nm irradiation, IC mg/mL on 4 Gy X-ray irradiation, and IC mg/mL on NIR λ = 1064 nm and X-ray irradiation)	[133]
Ga	8–20 nm	An anti-cancer agent based on gallium nanoparticles combined with gamma radiation. Efficacy was confirmed by a study in female mice that had solid Erlich cancer. Gallium nanoparticles were biologically synthesized using Lactobacillus helveticus cells.	[36]
Ga	8–20 nm	A therapeutic agent in the form of gallium nanoparticles combined with low levels of gamma radiation was used to treat hepatocellular carcinoma induced by dietary nitrosamine in rats. A strain of Bacillus helveticus was used to synthesize GaNPs.	[134]
Ga	5–7 nm	An agent that prevents hepatocellular carcinoma (HCC) from metastasizing to the brain by inhibiting BSSP4 mRNA expression, leading to suppression of multiple tumour growth factors. The study was conducted in rats.	[46]
Ga	GaNS: 220 nm GaNR: 255 nm LMNR: 237 nm Nanospheric, rod-like structures	A therapeutic agent in the form of liquid gallium nanoparticles, characterized by variable shape (from spherical to rod-shaped). During the synthesis, liquid metal sonication was used along with HS-PEG-HS to increase the stability of the system and biosafety in the bloodstream (spherical structure- GaNS). In addition, gallium- GANR nanorods and gallium- indium- LMNR alloy nanorods were synthesized. The nanoparticles have the ability to target tumours through specific binding between HA and overexpressed CD44 receptors on breast tumour membranes.	[40]
Fe/Ga	15–20 nm	Agents with potential therapeutic properties for hard and soft tissue cancers via hyperthermia. Magnetic Fe-Ga nanoparticles were synthesized using sol-gel polycondensation reaction.	[135]
Pd	10 nm 4 to 14 nm	Human Ovarian Cancer Cells (SKOV3). Potential agent for the treatment of ovarian cancer- SKOV3 cells. NPs were synthesized by treating palladium chloride with hesperidin.	[136]
Pd	Spherical in shape, 5–20 nm	IC50 300 nM for human breast cancer cells (MDA-MB-231) An agent with potential verpaeutic therapeutic properties, synthesized using saponin. Strong synergistic interactions have been demonstrated between PdNs, and trichostatin A (TSA) in cervical cancer cells.	[137]
Pd	30–153 flower shaped, size dependent on addiction of chitosan	The agent has been used for in vitro photothermal therapy and in vitro near-infrared photoacoustic imaging. Porous flower-shaped palladium nanoparticles were synthesized using chitosan and vitamin C.	[138]
Pd	In MV process: spherical particles, 11–33 nm	Antioxidant and Cytotoxic Effects Against Fibroblast-Like (HSkMC) 320 μg/mL and Human Lung Carcinoma (A549) Cell Lines (7.2 ± 1.7 μg/mL)	[139]
Pd	Cubic structure, an average size about 2–9 nm	Anti-cancer Activity Against MCF-7 Cell Lines	[140]
Pd	Various shapes, 3.1–6.5 nm	Reduced inherent cytotoxicity and high photothermal conversion capacity in the presence of NIR irradiation	[141]
Pt	55 nm, spherical	Photoacoustic/photothermal multimodal imaging at tumour sites	[142]
Pt/TPP	Flower shaped, 30–60 nm	Induction of cell death and G2/M-phase cell cycle arrest in human cervical cancer cells	[143]
Pt	Spherical shaped NPs with size ranges from 20 to 50 nm.	Cytoxicityactivity against MCF-7 cell line using PtNPs.	[144]
Se	Spherical shape, 25 nm,	Anti-cancer action and low toxicity to normal cells and its selectivity towards tumour cells	[145]
Sb	antimonene quantum dots coated with PEG, 2.8 nm, spherical shape	Notable NIR-induced tumour ablation ability	[146]
Sb	Sb nanopolyherdrons with PEG and 1-methyl-d-tryptophan, 65 nm, polyhedrons	A highly efficient photoacoustic-imaging-guided synergistic photothermal/immune-therapy of tumours in vivo	[147]
Sb	PEG coated antimoneny 4 nm, spherical nanosheets	Drug delivery, sensing, imaging, photothermal therapy and other cancer treatment fields	[49]
Sb	34–42 nm, spherical shape	Synergistic chemo—photothermal therapy	[148]
Sb	4 nm, spherical shape	NIR light-induced tumour ablation	[149]
Sb	Spherical particles, 1.6–2.9 nm	Infrared degradability of antimonene in tumour treatment	[66]
Sb	34–42 nm, spherical	Photosensitizer, DOX drug carrier degradable under NIR, modified with PAA, at a concentration of 200 mg/mL irradiation, and as a result of the release of the drug killed 97% of HeLa cells	[150]
Sb	2.8 nm quantum dots	PEG-modified photosensitizer; at a concentration of 200 mg/mL and the irradiation killed 90% of MCF-7 and HeLa cells)	[151]
Sb	Nanopolyhedrons	Drug/antibody carrier, photosensitizer, with photoacoustic properties modified by oleylamine, dodecylthiol, and DSPE-PEG2000 with activity confirmed against 4T1 cells LC85 62.5 µg/ml	[152]
Sb	2D and 3D nanostructures	Drug/antibody carrier, photosensitizer, with photoacoustic properties modified by 4T1 cell membrane action confirmed against 4T1 cells	[153]
Sb	2D nanostructures (237.1 ± 67.2 nm) and quantum spheres (164.3 ± 27.4)	Radiosensitizer that undergoes X-ray conversion to toxic Sb_2_O_3_, modified with PLGA with activity confirmed against A375 cells	[154]
Sb	140 nm × 4 nm; after modification, 90 nm × 6 nm	DOX drug carrier, photosensitizer with photoacoustic properties, stabilized with DSPE-PEG3000 (achieved 43.3–43.7 °C after NIR λ = 808 nm, 0.5 W/cm^2^ irradiation, 41.8% conversion; with confirmed effect against MCF-7 cells-upon irradiation and drug release killed 91.5% of cells; in mouse study 98% inhibition of tumour growth)	[155]
Sb	2.0 ± 0.6 nm; quantum dots, 150–200 nm after modification; spherical	Photosensitizer and drug carrier, stabilized and directed by modification of HS-PLGA-PEG-FA (obtained 40.0 °C after NIR irradiation λ = 808 nm, 1 W/cm^2^, 42.53% conversion; with confirmed activity against HeLa, MCF-7, HepG2, PC3 cells)	[156]
Sb	Nanoplatelets (52.52 × 20.24 × 15 nm;)	Contrast agent with photoacoustic properties and high photosensitizing potential (obtained 207.9 °C after NIR irradiation λ = 808 nm, 2 W/cm^2^, 42.36% conversion)	[49]
Se	130 nm	DOX drug carrier directed by transferase and stabilized by chitosan with proven activity against MCF-7, HepG2, A375 (IC50 7.1 to 11.1 μm)	[157]
Se	180 nm	RuPOP drug carrier directed folic acid with proven activity against HepG2 (IC50 0.33 ± 0.02 μm) and R-HepG2 (IC50 0.24 ± 0.02 μm)	[104]
Se	50–150 Sb nm	DOX drug carrier, with proven effect against MCF7 cells, a significant amount of cell killing was observed after 6 and 36 h at 100 μg/mL and 25 μg/mL, respectively; there was a marked improvement in effect against DOX drug alone	[19]

**Table 2:** Metal oxide nanoparticles used in anti-cancer therapies.

Material	Nanoparticles Parameters	Nanoparticles Functions	Ref.
Bi_2_O_3_	Spherical shape, 35 nm	Combination with photodytase for targetable phototherapy	[183]
Bi_2_O_3_	Spherical particles, 97 nm	Examined dose-dependent cytotoxicity and 23 apoptosis response of Bi_2_O_3_ NPs in human breast cancer (MCF-7) cells	[184]
Bi_2_O_3_	43 nm-α-B_2_iO_3_, 37 nm-α-Bi_2_O_3_ -APTMS (3-aminopropyl)trimethoxysilane	Theranostic system (diagnosis and treatment in one)	[80]
Bi_2_O_3_	Bi_2_O_3_ with hyaluronic acid, quasi-spherical shape, 45 ± 0.6 nm	Targeted computed tomography imaging and tumour radiosensitization	[79]
CeO_2_	15–20 nm	Cytotoxic effect against colorectal cancer cell line	[169]
CeO_2_	Band-gap was 4.1 eV, spherical shape, 15–20 nm	Cytotoxic effect against colorectal cancer cell line	[170]
CeO_2_	Calculated crystallites from Scherrer around 4 nm	Sensitization to radiotherapy	[185]
CeO_2_	Spherical particles, 100–200 nm	Cytotoxic effect on prostate cancer cells	[186]
CuO	Spherical particles average 5.4 nm	Sensibiliser for radiation therapy	[187]
CuO	Nanoparticles can be seen in TEM images, but the measured diameter was about 1–2 μm	Antitumour activity on human colorectal cancer cell lines	[188]
CuO	10 to 190 nm average 110 nm	Photothermal activity against lung cancer	[174]
Fe_3_O_4-_DOX	~22 nm, spherical, Au-Fe_3_O_4_, DOX	Magnetic gold nanoparticles modified with iron oxide nanoparticles and functionalized with PEG for transporting the anti-cancer drug DOX	[189]
Gd_2_O_3_	3–4 nm	MRI imaging	[178]
Gd_2_O_3_	Average size 1.8 nm	Electron capture anti-cancer therapy	[190]
Gd_2_O_3_	100 nm	Magnetic imaging and theranostics	[177]
GaO(OH)	Oval GaO(OH) with β-cyclodextrin NPs, 380–400 nm	A drug carrier	[159]
HfO_2_	65 nm	Anti-cancer effects Enhancing the effects of radiation therapy	[191]
SnO_2_	Irregular NPs, 21.3 ± 11.4 nm	Cytotoxicity, ROS and H_2_O_2_ generation along with lipid peroxidation, SnO_2_ NPs weakened the antioxidant capacity of cells	[192]
SnO_2_	Folic acid coated tin oxide nanoparticles (FA-SnO_2_ NPs), Spherical NPs, 157 nm,	Targeting human ovarian cancer cells with minimal side effects against normal cells	[2]
TiO_2_	TiO_2_ -PEG Spherical particles, 10–25 nm,	Energy conversion to enhance induced heat efficacy in photo-thermal therapy; reduction of melanoma tumour size after PTT	[193]
ZnO	Spherical and hexagonal NPs, 45–60 nm	Antioxidant and cytotoxic properties in lung cancer (A549) cells	[166]
ZnO	Approximately 20 nm	Cytotoxic properties against human ovarian cancer cells	[158]
ZnO	Nanoparticles can be seen in TEM images, but the measured hydrodynamic diameter came out to 13.87 nm	Anti-cancer agents	[167]
ZnO	Marsdenia Tenacissima hexagonal, elongated and rod-like ZnO at different nanometre scales	Anti-cancer agent	[168]

**Table 3:** Layered double hydroxides (LDH) used in anti-cancer therapies.

Material	Nanoparticles Parameters	Nanoparticles Functions	Ref.
LDH	(Mg_0.68_Al_0.32_(OH)_2_(CO_3_)_0.16_·0.1H_2_O), hexagonal, 200 nm	Carrier of anti-cancer drugs and their targeting of lung cancer cells A549	[198]
LDH	(Mg_2_Al(OH)_6_(CO_3_)_0.5_·0.1H_2_O), 100 ± 25 nm	Anti-cancer drug carriers with targeting function through chemical conjugation with the ligand folic acid (FA)	[195]
LDH	Mg_2_Al(OH)_6_(NO_3_)·0.1H_2_O	Carrier of the anti-cancer drug methotrexate (MTX) in the control of osteosarcoma cells Saos-2 and MG-63	[201]
LDH	Mg_0.68_Al_0.32_(OH)_2_(NO_3_)_0.32_·0.1H_2_O	MTX anti-cancer drug carrier with low cytotoxicity tested on human breast adenocarcinoma MCF-7 cells	[202]
Cu-Al LDH	Shape of plates before modification	DOX anti-cancer drug carrier modified with *Plantago ovata* plant extract with antibacterial properties	[203]
Fe_3_O_4_/Zn-Al LDH	Large specific surface area of 121.7 m^2^/g	Carrier of anti-cancer drugs with controlled release and cytotoxic, magnetic properties, tested on human hepatocellular carcinoma cells HepG2	[22]

**Table 4:** Metal-centred complexes obtained and their anti-cancer activities.

Complex	Organic–Inorganic Compound	Metal-Ligand	IC 50 (Micromolar)	Ref.
NHC	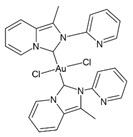	Au	5.2 (for A549)	[207]
NHC	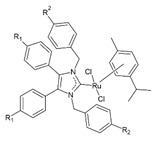	Ru	2,4-80 (for MCF7)	[208]
di-DACH-Pt(IV)-COOH	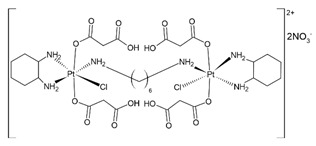	Pt	36.4-51.6 (for HCT-8)	[209]
dpphen	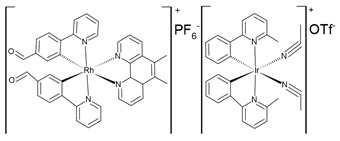	Rh/Ir	12,5 (for A375)	[210]
Phosphole 14	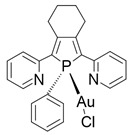	Au	5,4 (for NCH37)	[211]
17 marimastat	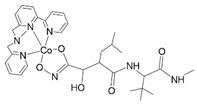	Co	0.9–1 (for MMP-9)	[212]
Eonate 21	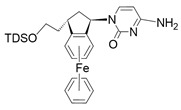	Fe	0,9-16,0 (for MCF-7)	[213]
Titanocene	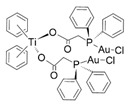	Ti/Au	3,2-8,7 (for A498)	[214]
Schiff base	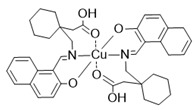	Cu	28,4 (for U87)	[215]
Chitosan	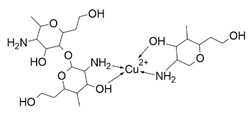	Cu	28,0 (for HCT15)	[216]

## Data Availability

The data presented in this study are available on request from the corresponding author.
